# Topology, robustness, and structural controllability of the Brazilian Federal Police criminal intelligence network

**DOI:** 10.1007/s41109-018-0092-1

**Published:** 2018-08-24

**Authors:** Bruno Requião da Cunha, Sebastián Gonçalves

**Affiliations:** 1 Superintendência da Polícia Federal no Rio Grande do Sul, Av. Ipiranga, 1365, Porto Alegre, RS, Brazil; 2 Instituto de Física, Universidade Federal do Rio Grande do Sul, Av. Bento Gonçalves, 9500, Porto Alegre, RS, Brazil

**Keywords:** Criminal networks, Modular networks, Netwrok robustness, Structural controllability

## Abstract

Law enforcement and intelligence agencies worldwide struggle to find effective ways to fight organized crime and reduce criminality. However, illegal networks operate outside the law and much of the data collected is classified. Therefore, little is known about the structure, topological weaknesses, and control of criminal networks. We fill this gap by presenting a unique criminal intelligence network built directly by the Brazilian Federal Police for intelligence and investigative purposes. We study its structure, its response to different attack strategies, and its structural controllability. Surprisingly, the network composed of individuals involved in multiple crimes of federal jurisdiction in Brazil has a giant component enclosing more than half of all its edges. We focus on the largest connected cluster of this network and show it has many social network features, such as small-worldness and heavy-tail degree distribution. However, it is less dense and less efficient than typical social networks. The giant component also shows a high degree cutoff that is associated with the lack of trust among individuals belonging to clandestine networks. The giant component of the network is also highly modular (*Q*=0.96) and thence fragile to module-based attacks. The targets in such attacks, i.e. the nodes connecting distinct communities, may be interpreted as individuals with bridging clandestine activities such as accountants, lawyers, or money changers. The network can be disrupted by the removal of approximately 2% of either its nodes or edges, the negligible difference between both approaches being due to low graph density. Finally, we show that 20% of driver nodes can control dynamic variables acting on the whole network, suggesting that non-repressive strategies such as access to basic education or sanitation can be effective in reducing criminality by changing the perception of driver individuals to norm compliance.

**Electronic supplementary material:**

The online version of this article (10.1007/s41109-018-0092-1) contains supplementary material, which is available to authorized users.

## Introduction

Despite recent efforts of Brazilian law enforcement agencies in combating organized crime, the horizon is not promising: homicide rates have spiked in 2014 reaching 29.1 deaths per hundred thousand people (Cerqueira et al. 2017), the country has become the second greatest consumer of cocaine in the world —turning into one of the most important corridors for international drug trafficking—, and corruption and money laundry have pervaded major enterprises and important political figures nationwide (UNODC 2015).

The problem is multivariate: from cultural and historical reasons to the structure of the Brazilian political, judicial, and law enforcement systems. At the same time, the sociological and behavioral literature supports both theoretically and experimentally the adoption of network methods in studying criminal rings (McGloin 2005; Sah 1991; Glaeser et al. 1996; Morselli 2003; Mastrobuoni and Patacchini 2012; Thornberry et al. 1993). These studies show that when a person is part of a social criminal network, some of his/hers individuality is lost, and the group starts acting as a whole. Therefore, attacking the structure of a criminal organization should block the clustering processes involved in the collective human behavior related to clandestine activities —this is precisely the aim of police, law enforcement and intelligence agencies. In this sense, several researchers have studied the structure and fragility of criminal networks (D’Orsogna and Perc 2015; Baker and Faulkner 1993; Krebs 2002; Reeves-Latour and Morselli 2017). For instance, the network structure and resilience of the Sicilian Mafia (often known as *Cosa Nostra*) was recently studied (Agreste et al. 2016). In that paper, the cooperation with Italian law enforcement agencies led to a bipartite network (contact and criminal), which showed different robustnesses to network attacks —the contact network is much more fragile to targeted attacks than the criminal one. However, the authors did not study the Mafia network’s modularity neither its robustness to important methods of network interventions such as the collective influence (Morone and Makse 2015) and the module-based attack (Requião da et al. 2015). Accordingly, other authors have studied Mafia syndicates, pointing to the strong hierarchical networked organization with a few *capi* (bosses) commanding the criminal activities (Cayli 2013). Furthermore, some papers have shed light into the modular structure of criminal networks either to detect non-trivial players in reconstructed phone call networks (Ferrara and et al. 2014) or to understand the internal structure of subgroups in a particular case study of a small local mafia group in Italy (’Ndrangheta) (Calderoni et al. 2017). Yet, little is known about how the modular nature of criminal networks affects its robustness to efficiently designed topological interventions.

Complete data concerning criminal or terrorist networks from reliable intelligence sources are usually unavailable or classified for legal and security purposes. As a result, researchers usually have to rely on public court data or news magazines, lacking uncut information. An example of such an approach is the study by Ribeiro et al. (2018) which analyzed unclassified data from daily newspapers of political corruption scandals in Brazil over the last two decades. In order to fill this gap, we introduce and share with the scientific community the intelligence (anonymized) data collected by the Brazilian Federal Police during 2013. The data correspond to federal crimes resulting in a web of almost 24,000 individuals (including the federal scandals studied in Ribeiro et al. (2018) that occurred before 2013). Such unique set of data is available thanks to an ongoing collaboration with the Brazilian Federal Police. It expands across a large amount of criminal relationships and illegal practices, allowing us to deeply study this criminal intelligence network structure.

From the network science point of view, there are two main aspects related to police interventions: the topological robustness of criminal networks and their flexibility or resilience to disruption. Topological robustness is a static problem related to finding the minimal set of nodes whose removal from the network would break it into many disconnected components with size not comparable to the original network (Morone and Makse 2015). Network flexibility (or resilience) is in turn a dynamic feature that indicates how a criminal network re-order itself in response to law enforcement interventions (Morselli and Petit 2007; Morselli 2009). Such flexibility is believed to be due to the replacement of arrested members of criminal groups that rapidly adapt to the tactics used by the police (Spapens 2011). In this sense, in a study of a drug-related network from the Dutch Police (Duijn et al. 2014), researchers discovered that criminal organizations may react to targeted attacks to its most central nodes by becoming more efficient or robust, contrary to the common sense. The positive counterpart is that targeted attacks diminish criminal networks internal security, leaving them more exposed to law enforcement and intelligence agencies. These results stress the importance of network interventions before criminal groups have the opportunity to re-organize and enhance their robustness to targeted attacks. Thence, even though criminal networks are dynamic in nature and very reactive to law enforcement operations, rapid periodical interventions should keep criminals from adapting in a stable way. This is precisely why it is paramount for the police to identify the minimal dismantling set for criminal networks. This is a second important feature of our contribution: to identify the most effective heuristic attack strategy to disrupt the Brazilian federal criminal intelligence network into many disconnected small fragments.

Nonetheless, crime can be approached not only through repressive means such as confrontation and imprisonment (Machin et al. 2011). Recent articles have explored the effects of illiteracy on crime (Alves et al. 2018), and once thought individualistic attributes are now known to spread through contagion mechanisms over social networks (Christakis and Fowler 2007; 2008; Fowler and Christakis 2008). In this sense, dynamical features such as education and literacy if controlled could act as proxies for decreasing violence and general delinquency. Therefore, one would be interested in understanding if it is possible to take a dynamical variable acting on a criminal network from an initial unwanted state to a desirable lower state by influencing the appropriate driver individuals. This is precisely the framework of mathematical control theory (Yuan et al. 2013). However, mostly due to the lack of data there is a deep gap in the literature concerning the controllability of criminal networks. This is another important issue we address thanks to the unique data we present, i.e. the controllability a real-world criminal intelligence network.

Accordingly, our contribution is fourfold. We first introduce a unique dataset gathered by the Brazilian Federal Police and share it with the network science community (the original data can be found in the supplementary material). After that, we study the Brazilian federal criminal intelligence network structure and robustness to targeted attacks. Finally, we explore the controllability of this network. We conclude the paper with a general discussion of the results and a perspective of future projects.

## Dataset

The data presented here are a subset of the database of records of criminal investigations conducted by the Brazilian Federal Police. It includes criminal investigation records of 23,666 out of 166,105 people, which is the current size of the database. The original purpose of the Brazilian Federal Police in designing this database was not to conduct scientific analysis, it was meant only to keep an intelligence record of criminals, suspects and their known real life relationships in hope that it could help in future investigations. Therefore, in the network science perspective the network was naively built in a simplified fashion by the police itself. In this sense, this is a typical Criminal Intelligence (CRIMINT) (Ratcliffe 2009; Brown 2007) database, and it consists of information gathered, collated, analyzed, recorded, reported and disseminated by the Brazilian Federal Police concerning identified criminals and known suspects. The relationship data differ from public and court data or information. It consists of information interpreted by federal officers using methods and techniques that led investigators to assign criminal responsibility and liaison among individuals. For security and legal reasons these techniques are classified. In this sense, the Brazilian Federal Police built its own criminal relationship network according to the assessment of each federal agent. Thence, individuals in this network are known criminals and suspects, not necessarily tried and convicted, since this a CRIMINT database. The relationship network was also built directly by the Brazilian Federal Police, and two individuals would be connected through an undirected edge if there were an intelligence report filled by a federal officer assigning real life co-participation in a federal investigation. Therefore, when building the network, even though there might be great differences among the relationships, the Brazilian Federal Police considered all of them as of the same type. We were later granted access only to the raw anonymized and cyphered edge list of this undirected and unweighted CRIMINT relationship network. Therefore it was and is not possible for us to build the relationship network in a different fashion (e.g. with directed or weighted edges) or to analyze features such as the distribution of the types of crime. The network is typically dynamic since the relationships were created and/or deleted during the period from April to August 2013 (five months time span). However, we were only granted access to a snapshot of the relationships cumulatively collected at the end of August 2013, thence temporal aspects could not be analyzed by us.

The original police database, in particular the subset at the time of the query, included classified information. On account of that, the data were filtered and anonymized by the Brazilian Federal Police prior to the release for scientific, academic, and collaborative purposes, in order to comply with legal and security requirements. In this sense, we didn’t have access to metadata that were further classified by the Federal Police and are, therefore, not presented here. Only the topological features of the relationships were preserved in order to study the adjacent network structure. The anonymized network data are available in the Additional file 1.

The investigations cover most Brazilian federal crimes. Nonetheless, the definition of federal crimes vary depending on time and on the legal system of each country. In Brazil, the legal set that defines crimes investigated by the Brazilian Federal Police is highly intricate (Brazilian Constitution 1988; Brazilian Federal Law 2002; Brazilian Penal Code 1940; Brazilian Electoral Code 1965; Brazilian Federal Law 1986). However, crimes included in this dataset are focused on the following illegal activities:

- drugs and arms trafficking, smuggling and misplacement;
- interstate organized bank robbery;
- online sexual predators of children;
- federal corruption;
- environmental crimes and crimes agains historical heritage;
- crimes against the social security system;
- counterfeiting;
- crimes against the elections;
- crimes against the financial system;
- fraud against federal institutions;
- money laundering related to the above crimes.

**Fig. 1:**
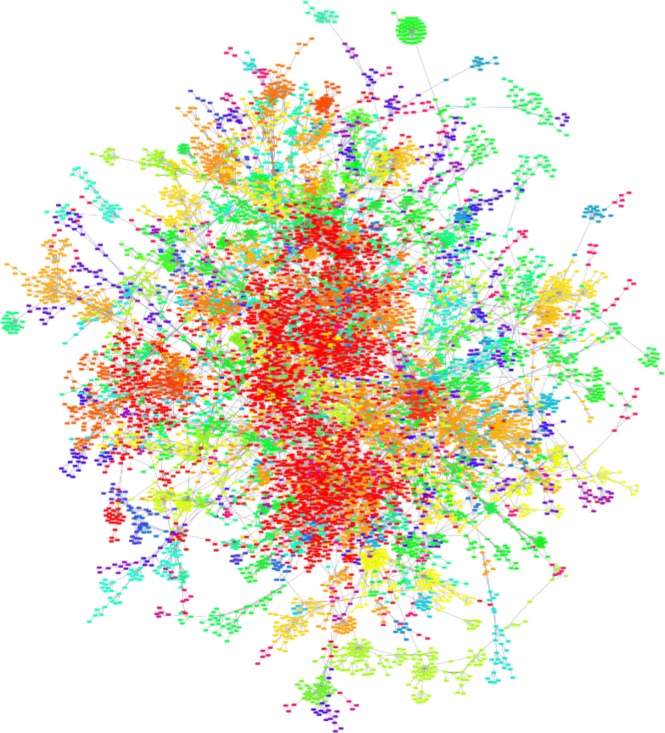
Representation of the largest connected component of the federal criminal intelligence network consisting of 9887 individuals. The colors are used to represent the different communities to which individuals are assigned according to the Louvain method (Blondel et al. 2008). As the number of modules is relatively large (91), several tones are perceived as the same color

The resulting undirected and unweighted network has *N*=23,666 nodes and *E*=35,913 edges distributed among 3425 unconnected components with an average size of only 7 individuals. However, the degree dispersion 〈*k*^2^〉/〈*k*〉=7.42 is much higher than the Molloy-Reed criterion which means that there is a giant component pervading the whole network (Dorogovtsev and Mendes 2013). This is remarkable for it was not expected that a giant component would rise in a set of actors committing criminal actions not related in principle to one another. Therefore, we focus only on the giant component of the network since the largest connected component may represent a generalized and self-organized criminal phase, more dangerous from a national security point of view. Such structure should be of concern for federal and national law enforcement, and intelligence agencies as well. The largest connected component consists of *N*=9887 nodes and *E*=19,744 edges (40% of the total number of nodes and 54% of the total number of edges, see Fig. 1). By defining <*k*>=2*E*/*N*, the average degree results in <*k*>≃3.994. From here we refer to this component by the name BFP2013 or by the expressions criminal intelligence network, federal criminal intelligence network or Brazilian federal criminal intelligence network.

## Criminal intelligence network structure

The density of a network, i.e. the number of edges as a fraction of the possible number of edges (*δ*=2*E*/*N*(*N*−1)), is usually related to its “brightness” (Klerks 2000; Toth et al. 2013). In the case of a criminal network, it gets more exposed as it gets brighter. In other words, in a bright network with a large number of connections among criminals, the investigation or capture of one actor, would help the authorities to extract critical information about the network structure (Duijn et al. 2014). A darker network, however, while hiding the structure from investigations, would slow down the transfer of information within the network due to the longer paths among criminals. However, even though covert networks tend to operate concealing their activities, their economic driven nature requires efficient communication to exchange money, goods, and merchandise for instance (Morselli et al. 2007). Topologically speaking, the network efficiency quantifies the exchange of information across the entire system and might be defined for a given graph *G* by the expression:

1 $$ \eta(G)=\frac{1}{N(N-1)}\sum_{i<j\in G}\frac{1}{d_{ij}},  $$

where *d*_*ij*_ is the distance between vertices *i* and *j*, and *N* is the total number of nodes. This metric spans both isolated components (*η*=0) and complete graphs (*η*=1) as it reflects how the actors in the network can communicate by measuring the smallest distance between vertices of the whole system (Memon and Larsen 2006).

**Fig. 2:**
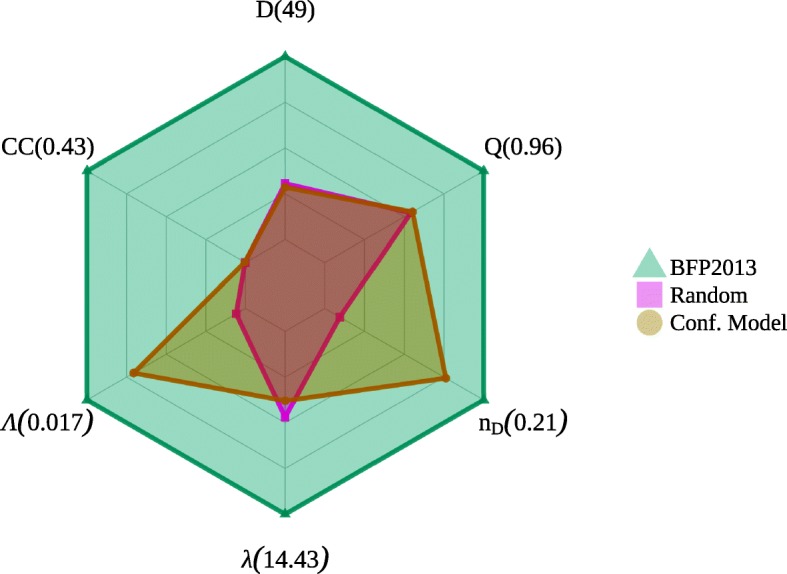
Radar chart displaying network parameters for the BFP2013 network (blue pattern and triangle symbols), its randomized counterpart (red pattern and square symbols), and the corresponding configuration model (yellow pattern and round symbol). Data for BFP2013, randomized, and configuration model networks, in this order, are the following: diameter (*D*=49,15,14), average shortest path length (*λ*=14.43,6.78,5.48), assortativity (*Λ*=0.017,0.001,0.012), average clustering coefficient (<*CC*>=0.430,0.001,0.002), modularity (*Q*=0.96,0.52,0.53) and number of driver nodes (*n*_*D*_=0.21,0.02,0.16)

Therefore, network density and network efficiency inform us about the compromise between security and effective diffusion of information and data, and this balance affects directly the network structure of criminal system (Baker and Faulkner 1993). Precisely, the network of the present study (Fig. 1) is “darker” than traditional social networks, i.e. it has low edge density, but, at the same time, it has low graph efficiency (see Table 1). The radar chart of Fig. 2 shows the topological features of the criminal intelligence network, a randomized version of it, i.e. after random rewiring all its edges but keeping *N*, *E*, and <*k*> the same, and the corresponding configuration model, i.e. a model that assigns degrees to vertices and then creates stubs, later it connects the stubs randomly, keeping the degree distribution intact creating a random graph in which the degree sequence is given (van der Hofstad 2016).

**Table 1:** Comparative data between the federal criminal intelligence network and other social networks: number of nodes (*N*), number of edges (*E*), edge density (*δ*=2E/N(N-1)), graph efficiency (*η* as defined in Eq. 1), and fraction of driver nodes *n*_*D*_ for the following social (communication, business, friendship and criminal) networks (Kunegis 2013): an e-mail communication network at the University Rovira i Virgili (U. Rovira i Virgili); a person-company leadership network (Corporate leadership); a Jazz musicians collaboration network (Jazz musicians); a gift-givings network between households in a Papuan village (Taro exchange); the well-known Zachary karate club network (Zachary karate club); a friendship network between boys in a highschool in Illinois (Highschool); a friendship network from hamsterster.com (Hamsterster); the network of suspected terrorists involved in the train bombing of Madrid on March 11, 2004 (Train bombing); a criminal dataset recorded by St. Louis Police in the 1990s (Crime); and the BFP2013 network

Type	Networks	Reference	N	E	*δ*	*η*	*n*_*D*_
Communication	U. Rovira i Virgili	(Guimerà et al. 2003)	1133	5451	0.0085	30.0*%*	0.04
Business	Corporate leadership	(Barnes and Burkett 2010)	24	99	0.3587	63.5*%*	0.08
	Jazz musicians	(Gleiser and Danon 2003)	198	2741	0.1406	51.3*%*	0.03
Friendship	Taro exchange	(Hage and Haray 1983)	22	78	0.1688	48.8*%*	0.04
	Zachary karate club	(Zachary 1977)	34	78	0.1391	29.4*%*	0.29
	Highschool	(Coleman 1964)	70	366	0.0758	44.7*%*	0.09
	Hamsterster	(Hamsterster full network dataset – KONECT 2017)	2426	16,631	0.0056	20.8*%*	0.30
Criminal	Train bombing	(Hayes 2006)	64	243	0.1205	44.8*%*	0.19
	Crime	(Crime network dataset – KONECT 2017)	829	1473	0.0043	21.5*%*	0.17
	BFP2013 (Fig. 1)	Dataset section	9887	19,744	0.0004	08.4*%*	0.21

The data highlight that the criminal intelligence network has a complex, non-trivial structure far from being random. The high average clustering coefficient (< *CC* > =0.43) associated with its average shortest path length (*λ*=14.43), as compared to its randomized counterparts (simple rewiring and configuration model respectively, <*CC*> =0.001,0.002 and *λ*=6.78,5.48), points out to the small-worldness (Dorogovtsev and Mendes 2013) feature of BFP2013. Accordingly, Humphries and Gurney (Humphries and Gurney 2008) showed that a network is said to be a small-world network if $S^{\Delta }=\frac {<CC>}{<CC_{rand}>}\times \frac {\lambda _{rand}}{\lambda }>1$, where the first term corresponds to the ratio between the clustering coefficient of the network and of its randomized version, and the second term is the ratio between the average shortest path length of the randomized network and of the original graph. Specifically in the present case, *S*^*Δ*^∼202 and 82 for simple rewiring and configuration model respectively, while the expected value of the linear fitting with network size observed in Humphries and Gurney (2008) is around 157, confirming the small-world nature of the BFP2013.

**Fig. 3:**
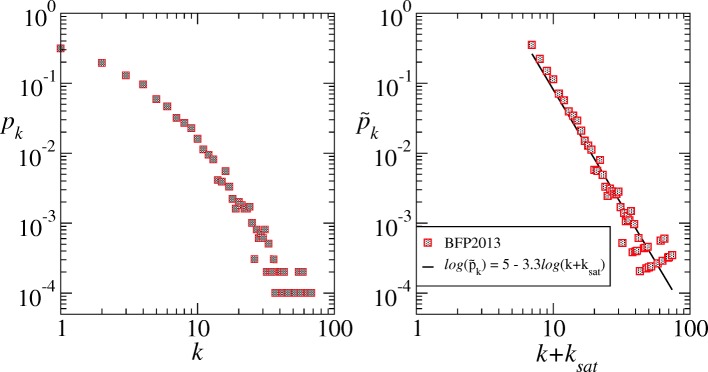
Degree distribution *p*_*k*_ for the federal criminal intelligence network in log-log axis, showing the typical real networks features of low degree saturation and high degree cutoff (left panel). Re-scaled degree function $\tilde {p_{k}}=p_{k} exp(k/k_{cut})$ as a function of $\tilde {k}=k+k_{sat}$ in log-log scale and the power-law fit (right panel). The linear fit is such that $log(\tilde {p})=(4.9643\pm 0.4147)-(3.2907\pm 0.1218)log(\tilde {k})$ with adjusted *R*^2^ of 0.937 and KS p-value 0.8693, meaning the model explains the data variability and that the null hypothesis is most probable. To reach this statistics we have proceeded according to the recipe for analyzing power-law distributed data proposed by Clauset et al. (2009). That is, we have proceeded a linear regression with the method of maximum likelihood in the log-transformed data to estimate the power-law parameters. The resulting statistics gives a very high p-value (0.8693) meaning the generalized power-law is a highly plausible hypothesis for the data. We then compared the result with a log-normal distribution (p-value 0.5441) via likelihood ratio test which resulted positive indicating that the generalized power-law model is favored over the log-normal. Simple power-law and exponential functions also do not show significant results

The degree distribution of a graph gives important clues about the nature of the network it represents. For instance, networks with homogeneous degree distributions, where the probability *p*(*k*) that an arbitrary node has degree *k* decays exponentially for large values of *k*, face a transition from a fully connected to a disconnected phase when a fraction *q*_*c*_ is randomly removed from it (Barabási 2016). While graphs in which *p*(*k*) has a heavy-tailed distribution are usually robust to random failure of nodes, but weak to targeted attacks to its most central nodes or hubs (Barabási 2016). Examples of networks with heavy-tailed degree distribution include the Internet, the World Wide Web, and in general most (large-scale) social networks (Dorogovtsev and Mendes 2013). In these cases, the degree distribution sometimes follows a power-law in a scale-free regime (*p*(*k*)∝*k*^−*γ*^, with 2<*γ*<3) and usually reveals generative models associated with preferential attachment, optimization, multiplicative models among others (Mitzenmacher 2004). However, real networks scarcely display pure power-law distributions. In general, two competing phenomena are present: low degree saturation and high degree cutoff (Barabási 2016). Usually, the number of low degree vertices is smaller than expected by a pure power-law regime due to an initial attractiveness of every node. The second behavior indicates a rapid drop in *p*(*k*) for *k*>*k*_*cut*_ due to inherent limitations in the number of edges each hub can accept. For typical social networks, this constraint is strongly related to the human limitation of maintaining more than 150 strong ties (a feature known as Dunbar’s number) (Gonçalves et al. 2011). Nonetheless, in the criminal case, besides this cognitive restriction, the high degree cutoff is also because of the lack of trust among criminals which is necessary to hide the network’s illegal activities, decreasing its brightness. We call this phenomenon the “no trust among thieves” effect. Illegal activities need to remain concealed from law enforcement investigations and this means that criminal contacts (relationship, conspirators, accomplices etc) need to be restricted (Morselli 2009). Therefore, trust and reputation are paramount in criminal cooperation in order to decrease the risk of the whole illegal operation being busted by the Police (Kleemans and Van de Bunt 1999; Von Lampe and Johansen 2004). On the other hand, when in time of operationalizing a given criminal agenda (taking action in a bank robbery for example), levels of trust could increase momentarily specifically in low level operational individuals (Erickson 1981). However, we do not have the dynamic data needed to analyze this burst of trust proposed in earlier researches, and our static results support the necessary lack of trust among criminals in the long run. This result also introduces an important empirical quantity for the “safe” level of lack of trust among criminals that has direct impact on the high degree cutoff of the degree distribution at approximately 60 relationships. Such effect, that reflects an embedded nature of criminal intelligence networks, could be used in future researches to model generative and agent-based models for criminal networks, for example. When both effects (cognitive and trust restrictions) occur there is a strong decrease in *k*_*max*_ which impacts the high degree cutoff. Power-law distributions with low degree saturation (*k*_*sat*_) and high degree cutoff (*k*_*cut*_) are usually fitted to

2 $$ p(k)\propto (k+k_{sat})^{-\gamma}\exp{\left(-\frac{k}{k_{cut}}\right)},   $$

which can be rewritten as a typical power law $\tilde {p}_{k}\propto \tilde {k}^{-\gamma }$, with the appropriate set of transformations $\tilde {p}_{k}=p_{k}exp\left (\frac {k}{k_{cut}}\right)$ and $\tilde {k}=k+k_{sat}$ (Barabási 2016). Figure 3 shows the degree distribution of the criminal intelligence network in log-log scale (left panel) and the re-scaled version of it according to the Eq. 2 (right panel), where we have used *k*_*sat*_=6 and *k*_*cut*_=60, resulting in an effective *γ*=3.29 (see Fig. 3 for statistics). With such transformation we recover the scale-free property of BFP2013.

The assortativity (*A*), i.e. the bivariate correlation between the degrees of connected nodes (Newman 2002), is another important aspect of a network. For instance, in the case of random networks, the correlation is zero in the limit of large graphs since edges are linked to each other independently of vertex degree. In assortative networks (*A*>0), nodes tend to connect to others with a similar degree, while in disassortative networks (*A*<0), high degree vertices tend to attach to low degree nodes. In social and business networks, highly connected people tend to relate to others with similar popularity in search for success, reputation, and social status (Newman 2002; 2003). Apparently, the same goes true with criminal networks which can be thought of a particular case of business networks. However, the value of *A* for the studied network, *A*=0.02, is very small, unraveling a close to neutral assortativity. A possible explanation could come from the maximum degree (*k*_*max*_=68) of the BFP2013 network which is much smaller than the structural cutoff for simple graphs (*k*_*s*_∼(〈*k*〉*N*)^1/2^=185.47) (Boguñá et al. 2004). Therefore, there are not enough edges to generate high levels of assortativity. As aforementioned when discussing the high degree cutoff, we propose that the reason why the maximum degree is much lower than expected lies both on cognitive restrictions related to Dunbar’s number and on the “no trust among thieves” effect needed to keep the network clandestine.

In hierarchical networks, the local clustering coefficient can be expressed as a function of the degree as (Dorogovtsev et al. 2002):

3 $$ CC(k)\sim k^{-\beta}.  $$

**Fig. 4:**
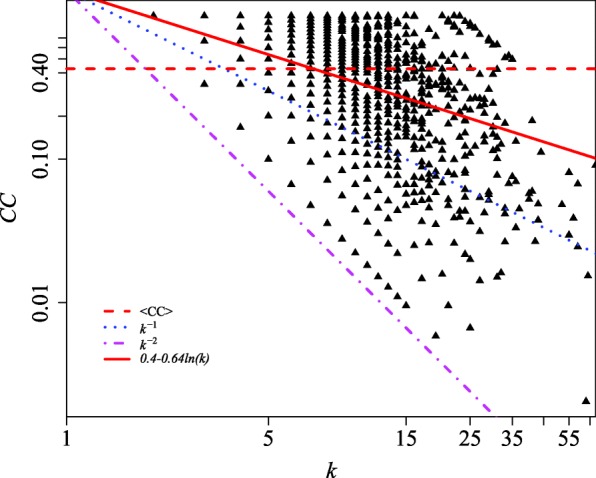
Clustering coefficient (*CC*) as a function of the degree (*k*) in log-log representation. The horizontal dashed red line is the average clustering coefficient (<*CC*>∼0.43). The dotted blue line is *CC*∼*k*^−1^, the dotdashed magenta line stands for *CC*∼*k*^−2^ and the solid red line is the linear fit *ln*(*CC*)=(0.40195±0.02096)−(0.63820±0.01287)×*ln*(*k*) with adjusted *R*^2^ of 0.2923 and very small p-value, meaning that the model explains only a few of the variability of the data around its average

It has been shown in previous works that *β*∼1 for deterministic scale-free networks as well as in a variety of real networks (Ravasz and Barabási 2003). To measure the topological hierarchy level of the BFP2013 network we display in Fig. 4 the clustering coefficient as a function of the degree in log-log scale. As that figure shows in detail, even though there is a strong saturation around high values of the clustering coefficient, *CC*(*k*) scales as *k*^−0.64^, indicating that the network is in fact hierarchical. As a mater of fact, low-*k* criminals (such as operatives) tend to have been identified in a small number of common investigations, i.e. many of them share neighbors that are usually higher profile criminals (such as local commanders) in a much smaller amount. This behavior results in high clustering coefficients. Complementary, high-*k* criminals were mostly investigated in many distinct law enforcement operations, but with different partners involved each time. These bridge-like criminals (such as bosses and *capi*) act as proxies for distant regions of the network, decreasing its diameter. Therefore, their neighbors are usually not connected among them, resulting in low *CC*(*k*) at high values of *k*. However, in Fig. 4 we observe a dispersion of *CC*(*k*) around high values of *k*, which might be due to the tendency of criminals to group with others with similar reputation.

## Network disruption and police intervention

The network provided by the Brazilian Federal Police consists of a static snapshot of CRIMINT relationships, and we do not currently have access to network dynamics. However, as discussed in the introduction, network robustness and resilience are two important features when dealing with criminal networks. Social and criminal networks are usually dynamic in nature with nodes and edges being created and deleted with time. As showed by Duijn et al. (2014), the attack to a set of criminal nodes might result in new connections that might reinforce network robustness. In fact, after a given perturbation (network attack) the system takes a characteristic time *τ* to reach a new equilibrium phase. Therefore, if the police could launch sequential attacks to the criminal network before *τ*, the network could be treated as static, and its dynamic and resilience features could be neglected. This is why robustness as a static problem is of paramount importance to police interventions. Even though with the data at hand we cannot know the characteristic time of resilience of the BFP2013 network, knowing its robustness at a given time might reveal important structural informations about it.

In this sense, we now study BFP2013 robustness to a series of targeted attacks. The attack on a network can be performed either by removing its nodes or by removing its edges (but keeping the nodes). In a topological perspective, node removal is always more effective in atomizing complex networks causing more damage per elimination than edge removal, since the deletion of a single node from the network results in the elimination of all the links attached to it (Iyer et al. 2013; Crucitti et al. 2004). Sometimes the traditional interpretation of topological interventions to criminal networks identifies the imprisonment of an individual as node removal (Morselli 2009). This usually holds true when considering isolated criminal groups or mafia rings. However, when dealing with CRIMINT networks such as BFP2013 which comprises relationships among distinct groups (such as drug lords and pedophiles for instance) from all crimes investigated by the Brazilian Federal Police, we believe the interpretation should change to reflect the bigger picture. For example, when a drug trafficking ring is dismantled by the Brazilian Federal Police, the criminals involved (nodes) do not get deactivated from the network. On contraire, they remain active in the CRIMINT network only losing (temporarily) some of its trafficking connections (edges), maintaining their corruption or sexual abuse relationships for instance— in real prison systems the individual should also increase its connectivity by imprisonment. In this sense, law enforcement operations are aimed at identifying and arresting criminals (nodes), which in turn may result in the elimination or at least in the temporarily suspension of some of its connections (edges), and not in the elimination of the individual from the CRIMINT network. Therefore, the deletion of a node in a CRIMINT network means the complete removal of the individual, which only occurs in the case of death or by total re-socialization of the subject. Nonetheless, it should be noted that this is a very simplified version of the real networked system analyzed, and in the BFP2013 case the network consists of only a static snapshot of a much complex phenomenon. However, considering only this simplified model of the criminal intelligence system, the aforementioned topological rationale might have important implications. From a network science point of view, re-socialization (e.g. by education or by work) should be in general a more effective strategy to fragment the criminal intelligence network than imprisonment since the first approach relates topologically to node removal while the second to edge removal. Still, considering all limitations of this model, analysis, and rationale, and bearing in mind one does not know how such system responds dynamically to topological disruptions, the death of key individuals (node attack), a strategy architecturally more efficient than edge removal, should reach the same results as re-socialization, *ceteris paribus*. This concept fits the distinction between Criminal Law of the Enemy (*Feindstrafrecht*) and Criminal Law of the Citizen as proposed by Günther Jakobs in 1985 (Jakobs 2010), in which certain people, as enemies of the society, should not have full protections of the civil and penal laws to protect this same society from systemic dangers. Jakobs proposes philosophically that when a recidivist criminal ignores all societal norms on behalf of its own criminal clan purposes he/she would be terminating the Hobbesian social contract, and in turn would enter a lawless natural state, losing his/hers civil rights, therefore turning into an enemy and losing its statue as a citizen (Jakobs 2010). However, we would like to strongly stress that even though such legal concept has been notably used in terror fighting (the idea of Taliban unlawful combatant for example), it is opposed and severed criticized by most scholars of penal law and legal philosophy (Negt 2014). From the topological point of view, once again considering all limitations of our analysis, perhaps the best argument against *Feindstrafrecht* is the fact aforementioned that re-socialization, a much more defensible strategy ethically, should reach the same network disruption effect.

**Fig. 5:**
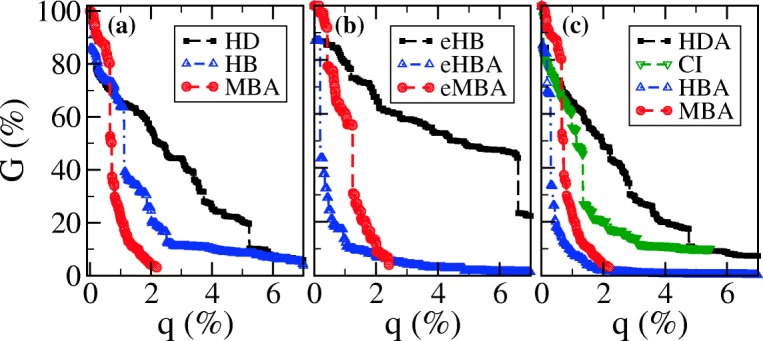
Fragmentation results of the federal criminal intelligence network represented by the relative size of the largest connected component, *G*, as a function of the percentage of nodes/edges removed, *q*, according to procedures based on different measurements: **a**, node removal by Degree (HD - black squares), Betweenness (HB - blue triangles), and Modular (MBA - red circles); **b**, edge removal by Betweenness (eHB - black squares), Betweenness Adaptive (eHBA - blue triangles), and Modular (eMBA - red circles); **c**, node removal by Degree Adaptive (HDA - black squares), Collective Influence (CI - lower green triangles), Betweenness Adaptive (HBA - blue triangles), and Modular (MBA - red circles)

To simulate the attacks on the BFP2013 network, we now perform node and edge attacks to the giant component as it was anticipated. We do that considering two different kind of strategies: high centrality attacks, when nodes or edges are deleted according to a list previously ordered by a chosen centrality index, and high centrality adaptive attacks when the list is iteratively ordered by a centrality index updated after each removal (Barabási 2016). Following these two types of strategies we test the network structural fragility against several procedures based on different centrality measurements (see Fig. 5): node-based High Degree Adaptive (HDA), High Betweenness Adaptive (HBA), High Degree (HD), High Betweenness (HB), Collective Influence (CI), and Module-Based (MBA) attacks, and edge-based High Betweenness Adaptive (eHBA), High Betweenness (eHB), and Module-Based attacks (eMBA).

The degree centrality is the number of connections a node has, while the betweenness centrality measures the fraction of shortest paths connecting two nodes that include the given vertex in its way (Iyer et al. 2013). The collective influence of a node takes into account the degree of its neighbors at a given distance *l* from it in the following way:

4 $$ CI_{\ell}(i)=(k_{i}-1)\sum_{j\in \partial Ball(i,\ell)}(k_{j}-1)\,  $$

where *k*_*i*_ is the node’s degree and the *∂Ball*(*i*,*ℓ*) is the set of all nodes at a distance *ℓ* from node *i*. The method of network fragmentation based on iteratively removing nodes with the highest collective influence *CI*_*ℓ*_ was proven to generate an attack list very close to the minimum dismantle set (Morone and Makse 2015), i.e. the minimal set of nodes that if removed would break the network into non-extensive components. The Module-Based attack (Requião da et al. 2015) is based on the modular nature of real networks, i.e. the tendency of complex networks to group into clusters densely connected internally but weakly connected among them. The density of internal (community) links when compared to the average density of edges is measured by the network’s modularity, *Q*, which ranges from − 1 to 1, and depends slightly on the community extraction algorithm used (Girvan and Newman 2002). Highly modular networks are fragile against MBA attacks, as we recently showed (Requião da et al. 2015). In fact, criminal relationships are expected to be organized in networks with a clear modular fingerprint. The reason is that weak connections among communities would favor network obscurity while the higher density inside the communities helps to run a business efficiently. Indeed, the present network has a very high modularity either using Louvain (Blondel et al. 2008) (*Q*=0.96) or using Infomap (Rosvall et al. 2009) (*Q*=0.88) methods.

To quantify the effects of each disruption strategy on the BFP2013 network, we measure the size of the largest connected component relative to the network’s original size, *G*(*q*), as a function of the fraction of removed elements, *q*. As pointed out in earlier research (da Cunha and Gonçalves 2017), the generalized robustness of a network to a given attack strategy is given by the metric:

5 $$ R=\frac{1}{N(1-G_{min})}\sum_{q=0}^{q_{max}}G(q)\;,  $$

where *N* is the number of nodes in the network, *q*_*max*_ is the point at which the attack ends, and *G*_*min*_ is the value of the relative size of the largest connected component at *q*_*max*_.

As mentioned before, the Brazilian Federal Police database continued to grow after the snapshot we analyzed and contains currently almost 2×10^5^ vertices. It is unfeasible to compute many centrality lists, remarkably HBA attacks, for networks of this size or bigger. Therefore, in order for the Brazilian Federal Police to identify high topological profile targets in the future according to its growing database it is important to check for the best strategy considering the trade-off between robustness (*R*) and the time (*t*) needed to compute the attack list. In this sense, the performance of an attack is measured by the relation *P*=*t*^−1^×*R*^−1^, where *t* is the time taken to complete the procedure and *R* is the robustness (da Cunha and Gonçalves 2017).

**Fig. 6:**
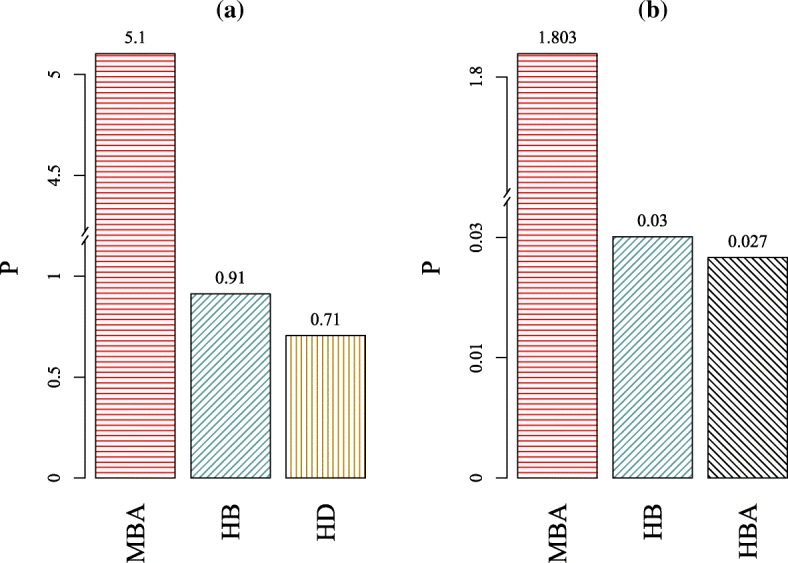
The histograms show the performance of the three best attacks on the federal criminal intelligence network. **a** shows Module-Based (MBA - horizontal red shades), High Betweenness (HB - inclined blue shades) and High Degree (HD - vertical golden shades) strategies for node removal, while **b** depicts MBA, HB and High Betweenness Adaptive (HBA - inclined black shades) methods for edge-based removal

In accordance with these considerations, the attack strategy with highest performance (see Fig. 6) is MBA both for node and edge attacks as expected for the network’s high modularity. However, the network is a little less robust to HBA, which in turn takes much more time to compute. Besides that, the BFP2013 is much weaker to HBA and MBA attacks than to the novel CI strategy as depicted in Fig. 5. In other words, the network would be fully atomized after removing approximately 2% of its vertices and almost 5% of its edges by HBA. The deactivation point at which all communities are detached from the core of the original graph is reached by the MBA prescription when nearly 2% of either its edges or nodes are removed. This means that even though node removal is in general more efficient than edge attacks, particularly in this network both strategies are very similar—for instance, the edge MBA has higher performance and similar robustness than the node HBA. To illustrate the effectiveness of HBA and MBA, the network would fragment completely by random attacks after the random failure of 80% of nodes or 86% of edges. From the criminal sciences perspective, one may say that re-socialization is, in general, a more desirable and sustainable strategy to lower crime levels than imprisonment. However, as far as the modular nature of BFP2013 is taken into account, both strategies show similar results mostly due to its low density of edges.

Recent studies (Ren et al. 2018a; 2018b) have suggested that a more realistic approach to network robustness would be to take into account a cost of removing nodes proportional to the number of edges deleted along with it. The authors argue that most state-of-the-art algorithms such as MBA or CI fail in efficiency when such a generalized network dismantling framework of node removal cost (such as node price, protection level or removal energy) is taken into account. Even though such framework may be applicable to a variety of real networks, we believe that it is not the case of criminal networks. For instance, high profile criminals that could, in principle, have higher levels of protection, violence or political influence should be precisely the ones targeted by the police. Likewise, since all edges in BFP2013 are criminal relationships, the mainstream criminal policy is precisely to eliminate the higher amount of them with the least number of actions– this is exactly the aim of confinement and prison, to remove the largest possible amount of exterior criminal edges of the inmate. We believe that such framework might be extremely suited to networks of networks when a criminal network functioning is dependent, for instance, on an infrastructure network or a non-criminal relationship network (e.g. family or friends). However, this is not the case of the BFP2013 network.

## Brazilian Federal Police network controllability

A dynamic system is said to be controllable if one can get it to evolve from any initial state to an arbitrary final state in a finite time by an appropriate choice of external inputs. However, for very large systems such as real networks it is more suitable to search for a minimum subset of nodes whose control guarantees mathematically control of the whole network. For instance, a dynamic variable such as opinion, wealth or general tendency to commit a crime evolving in time constricted to a criminal network topology is reducible to a minimum or zeroth level, at least in principle, if the network is controllable. Recently, it was developed the so-called structural controllability theory of directed networks (Liu et al. 2011), which consists of identifying a minimum set of individual driver nodes to achieve full control of complex networks, this framework was shown to be equivalent to a problem of maximum matching. More recently, it was shown that structural controllability can be achieved with a single time-varying input suggesting that nodal dynamics is the key factor in determining network controllability (Cowan et al. 2012). Nonetheless, the proposal is restricted to directed networks, which is not the case studied here. Therefore, the exact controllability theory (Yuan et al. 2013) is more suited to BFP2013. This framework is based on using the maximum geometric multiplicity of the adjacency matrix to find the minimum set of drivers required to fully control the network. In this sense, consider a linear system described by the following set of ordinary differential equations:

6 $$ \dot{\textbf{x}}=A\textbf{x}+B\textbf{u},  $$

where the vector **x** stands for the states of the nodes, *A* is the adjacency matrix of the network whose elements are *a*_*ij*_=1 if nodes *i* and *j* are connected and *a*_*ij*_=0 otherwise, **u** is the vector of controllers and *B* is the control matrix. The network represented by *A* is said to be controllable according to the framework of the exact controllability theory (Yuan et al. 2013) if we control a minimum fraction of nodes (called drivers or controllers) given by:

7 $$ n_{D}=\frac{1}{N}max\{1,N-rank(A)\}.  $$

In this sense, it was previously shown by Liu et al. that the degree distribution determines in great extent the controllability of the network (2011). For example, in the case of unweighted and undirected Erdös-Rényi networks, *n*_*D*_→0 for typical values of the connecting probability (Yuan et al. 2013). In their seminal article, Liu et al. (2011) have shown that, counter-intuitively, many social networks usually have very low *n*_*D*_ values when compared to biological or infrastructure networks. For instance, in the networks studied by Liu et al. (2011), gene regulatory networks display *n*_*D*_ as high as 0.96 (TRN-Yeast-1), while social networks have values as low as 0.04 (Slashdot), indicating that few individuals could in principle control the whole network. A similar behavior is shown here. In Table 1 we show *n*_*D*_ for many social networks, and the values range from 0.04 (in the communication network of University of Rovira i Virgili) to 0.30 (in the Hamsterster network). The BFP2013 network also shows low level of *n*_*D*_=0.21, suggesting that it could be controlled by only 2076 criminals out of 9887 individuals. This result supports the idea that criminality levels can be mitigated by non-repressive policies. For instance, supposing norm compliance (or obedience to law) is not an individual attribute, but a dynamic variable that spreads through the network by contagion mechanisms, according to these results it would be possible to change the whole network perception of breaking the law by flipping the behavior of approximately 20% of individuals. This rationale might suggest that mitigation of criminality can be achieved by non-repressive policies such as general access to basic education, sanitation or health, that in turn could change the perception of driver individuals to norm compliance.

## Conclusion

Thanks to a recent data acquisition program by the Brazilian Federal Police, we are able to study the network structure, robustness, and control of a snapshot of a large and unique criminal intelligence network covering different classes of federal crimes all over Brazil. The network was built directly by federal agents assigned to each investigation for intelligence and investigative purposes. The network was anonymized and cyphered before it was made available for us to study. In this paper, we share and analyze this unique network consisting of 23,666 individuals in 35,913 undirected and unweighted relationships. Surprisingly, the network consisting of initially distinct crimes such as drug trafficking and online children predators, has a giant component holding more than 40% of the nodes and 54% of the edges.

By focusing on this giant component (BFP2013) we show that the network has small-world and scale-free behaviors, being “darker” than traditional social networks, combining both low edge density and low network efficiency. These features are related to the clandestinity of the network that constantly tries to hide from law enforcement surveillance. The network also has a heavy-tailed degree distribution that is fitted to a generalized power-law with low degree saturation and high degree cutoff. The first phenomenon is due to the initial attractiveness of each node and the second, which explains the low maximum degree, is related to both cognitive limitations and to the “no trust among thieves” effect, i.e. criminals tend to have a reduced number of relationships in order to protect their illegal activities. This effect, which we believe reflects a subjacent nature of criminal networks, introduces an empirical quantity to a “safe” level of trust among criminals that might be used in future works to design generative or agent-based models for criminal networks consisting of multiple rings of distinct criminal actions such as the one studied here. This high degree cutoff also results in a close to neutral degree assortativity. The network is also highly hierarchical, a feature directly related to the behavior of the clustering coefficient, reflecting the fact that a few prominent individuals are responsible for network cohesion, while most low-k criminals participate only in a small amount of illegal enterprises with a repeated number of accomplices.

The criminal intelligence network is highly modular, which is a result of the compartmentalization of activities, i.e. the low density of connections among modules favor clandestinity while the higher fraction of edges inside communities enhances internal efficiency. Consequently, BFP2013 is highly weak to module-based attacks, being deactivated after the removal of approximately 2% of nodes (198 criminals) and 2.5*%* of edges (494 relationships). Although in general it is more efficient to remove nodes than edges, particularly in this network both strategies have similar results because of its low graph density. This is an important feature of this analysis. According to our interpretation of law enforcement topological disruptions of global CRIMINT networks, node removal corresponds to either re-socialization or death of key individuals. Edge removal, on its turn, has a close relation to imprisonment. *Ceteris paribus*, since mathematically removing nodes fragments general graphs faster than edge attacks, one could assert, as similar to Jakobs in his Criminal Law of the Enemy theory (Jakobs 2010), that the killing of key criminals would fragment the network in a more efficient way. Our results, however, introduce topologically two different approaches. For instance, re-socialization should have the same topological effect on fragmenting the criminal network. Besides, due to the low graph density of the BFP2013 network, the difference on the number of removals needed to fragment the CRIMINT network studied here in both node and edge removals approaches is very small (2% of nodes versus 2.5*%* of edges). This means in our rationale that the imprisonment of key topological criminals should reach very similar fragmentation levels as compared to the other two hypotheses, specially when compared to (*Feindstrafrecht*) theories that have an evident high social and ethical cost. We argue that the weakness of this network to targeted attack to its bridges among communities might have an important impact in law enforcement perception of high profile targets, i.e. criminal intermediates such as lawyers, accountants, black-market dealers, and money launderers operating for different groups have a structural role more prominent than the role of big bosses or *capi*. The biggest corruption scandal in the history of Brazil known as “Operação Lava-Jato” (Car Wash Operation in English) fits precisely this framework. It started as an international drug trafficking investigation by the Brazilian Federal Police in which a black-market money changer was later identified as the one responsible for laundering cash not just for drug lords but also for a highly intricate federal corruption scheme that lead recently to the conviction of former secretaries, congressmen, senators and presidents.

However, considering BFP2013 is a CRIMINT network it may not be possible to build legal cases in many situations. Therefore, one must consider non-repressive alternatives to police interventions, i.e. without the removal of nodes or edges. In this sense, we have shown that 2076 criminals out of 9887 individuals could in principle control the dynamic of linear systems evolving on this network. We suggest that this result indicates that criminality may be faced by non-criminal policies shaped to flip the perception of a small subset of driver individuals about the obedience to law. Nonetheless, in control theory one is usually interested in finding a stable final state or else the network will easily move away. Besides, in social networks the drivers are people and even the task of engineering a single input could raise ethical and legal issues. These are all question we will address in future contributions. Moreover, we plan to explore in the future how spread dynamics behave in controllable networks and to understand the role of superspreaders and superblockers in criminal networks (Radicchi and Castellano 2017).

It is important to note that our research focuses on a deliberately simplistic model of a criminal system with much of its complex structure not considered in order to obtain some insight about the simplified embedded topological mechanism of the network concisely. Therefore, our study has several limitations. First, the network consists of relationships identified by intelligence officers after a criminal intelligence cycle. This means that connections are manifold and may include relations such as contact, prison mates, co-offending, telephone or internet communications etc. However, the Brazilian Federal Police did not take into account the possibility of building a multiplex criminal network and the data were shared consisting of a condensed, simplified, undirected, and unweighted graph. Second, the BFP2013 consists of a static snapshot of a truly dynamic system that evolves in time and obviously responds to topological interventions. This highly limits our study of the CRIMINT network since we were not granted access to any temporal evolution of the system. All in all, even though this is only a simplified analysis of a much more intricate complex system, we believe the results we show in this paper might help understand some important underlying features of criminal networks, specially when considering the size, dimension and uniqueness of the criminal intelligence shared by the Brazilian Federal Police.

## Additional file

Additional file 1 This additional file consists of a CSV file with the anonymized edge list relative to the Brazilian Federal Police criminal relationship data, i.e. a 35,913-by-2 matrix. Each cell contains a hash which identifies an investigated person, and two adjacent cells indicate a criminal intelligence relationship between them. (CSV 2455 kb)

## Abbreviations

BFP2013: The giant component of the Brazilian Federal Police criminal intelligence network
CI: Collective influence attack
CRIMINT: Criminal intelligence
eHB: Edge-based high betweenness attack
eHBA: Edge-based high betweenness adaptive attack
eMBA: Edge-based module-based attack
HB: High betweenness attack
HBA: High betweenness adaptive attack
HD: High degree attack
HDA: High degree adaptive attack
MBA: Module-based attack
